# Supplementary material for: Combining Analytical Approaches and Multiple Sources of Information to Improve Interpretation of Diagnostic Test Results for Tuberculosis in Wild Meerkats
Source: Animals (Basel). 2021 Dec 4;11(12):3453. doi: 10.3390/ani11123453 (PMC8698085; doi:10.3390/ani11123453)
Supplement: Supplementary file 1 [file animals-11-03453-s001.zip › animals-1459951-supplementary/Supplementary Figures.pdf]

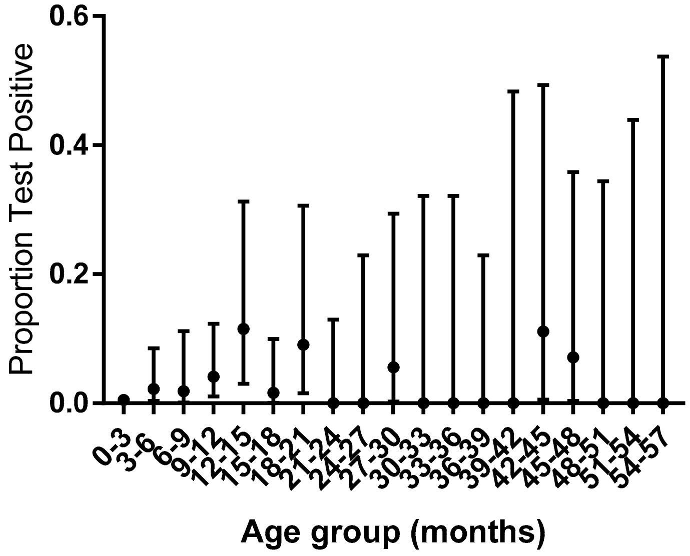

Figure S1. Relationship between serological test result and meerkat age. All 704 serology samples collected were classified as either positive or negative and assigned to a category based upon the animal's age at the sampling point. No association is seen between an animal's age, and its tuberculosis test status based upon serology.

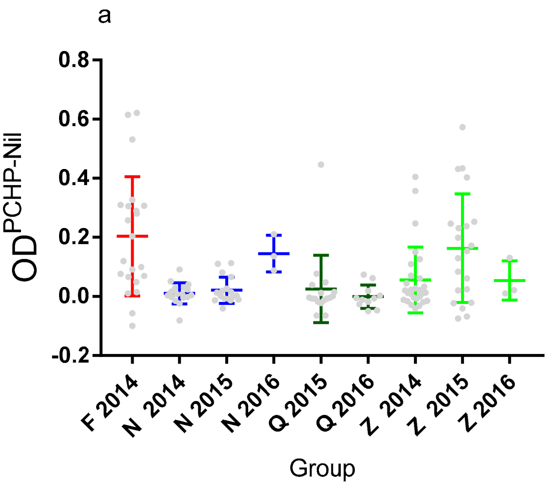

Figure S2. IFNγ Inducible-Protein 10 Release Assay results for individuals in 4 social groups (F, N, Q, and Z). Results are consistent within a group over the three years of testing, except for group N where a disease outbreak was observed in late 2016. Results are given for Optical Density OD) of the PCHP assay adjusted for background IP-10 ($OD^{PCHP-nil}$).
