# Supplementary material for: Combining Analytical Approaches and Multiple Sources of Information to Improve Interpretation of Diagnostic Test Results for Tuberculosis in Wild Meerkats
Source: Animals (Basel). 2021 Dec 4;11(12):3453. doi: 10.3390/ani11123453 (PMC8698085; doi:10.3390/ani11123453)
Supplement: Supplementary file 1 [file animals-11-03453-s001.zip › animals-1459951-supplementary/Supplementary Table.pdf]

Table 1. Survival analysis of time until the appearance of visual signs of tuberculosis. Univariable analysis examining the effect on the time to the observation of sub-mandibular lymph-node swelling in 126 meerkats. Clinical signs were observed in 22 of these animals. Serology was performed using the DPP assay, CMI using the described IPRA, and the culture variable is based upon culture of a tracheal wash sample. A positive status was conferred if one or more of the individual tests were positive. The Cox proportional hazards assumption was met for all models.

.		Hazard Ratio	95% Confidence Interval	Wald test p-value
Dominance	No			0.174
	Yes	0.46	0.15-1.41	
Sex	F			0.162
	M	0.54	0.22-1.29	
Age	<6 months			0.657
	6-12 months	0.73	0.18-2.93	
	>12 months	0.58	0.18-1.88	
Serology	Neg[a]			0.072
	Pos[b]	3.91	0.89-17.23	
CMI	Neg[a]			0.927
	Pos[b]	1.05	0.34-3.27	
Culture	Neg[a]			
	Pos[b]	*	*	*
Parallel	Neg[a]			0.708
	Pos[b]	0.83	0.30-2.26	

[a]Neg, Negative. [b]Pos, Positive. *No tracheal wash culture positive animals were found in this analysis.
